# ABCG2 protein expression in tumors of patients with non-resectable pancreatic cancer treated with gemcitabine and *nab*-paclitaxel

**DOI:** 10.3389/fonc.2025.1558184

**Published:** 2025-06-06

**Authors:** Susy Shim, Mette Bak Nielsen, Mikkel Eld, Jan Stenvang, Rasmus Froberg Brøndum, Britta Weber, Anne Krejbjerg Motavaf, Morten Ladekarl

**Affiliations:** ^1^ Department of Oncology and Clinical Cancer Research Center, Aalborg University Hospital, Aalborg, Denmark; ^2^ Department of Clinical Medicine, Aalborg University, Aalborg, Denmark; ^3^ Department of Pathology, Aarhus University Hospital, Aarhus, Denmark; ^4^ Department of Pathology, Aalborg University Hospital, Aalborg, Denmark; ^5^ Department of Drug Design and Pharmacology, Copenhagen University, Copenhagen, Denmark; ^6^ Scandion Oncology A/S, Copenhagen, Denmark; ^7^ Center for Clinical Data Science, Aalborg University and Aalborg University Hospital, Aalborg, Denmark; ^8^ Department of Oncology, Aarhus University Hospital, Aarhus, Denmark

**Keywords:** gemcitabine, *nab*-paclitaxel, ATP-binding cassette protein, ABCG2, BRCP, chemotherapy resistance, progression-free survival, immunohistochemistry

## Abstract

**Background:**

ATP-binding cassette (ABC) proteins are transmembrane efflux pumps that play a role in Multi Drug Resistance. ABCG2 and ABCB1 have been suggested as important mediators of resistance to chemotherapy (CTx) in pancreatic cancer (PC). We determined the expression of ABCG2 and ABCB1 proteins in PC and the impact of ABCG2 on outcome of treatment with gemcitabine and *nab*-paclitaxel (GemNab).

**Materials and methods:**

140 patients with sufficient tissue for assessment that had initiated palliative treatment with GemNab for non-resectable PC from 2011 to 2019 were included at two institutions. From achieved tissue, new sections were cut and stained for ABCG2 and ABCB1. Staining was evaluated by consensus of maximum score by two pathologists. Progression-free survival (PFS) was the primary endpoint.

**Results:**

ABCB1 expression was observed in only one case (0.7%). ABCG2 was expressed in 33% but more frequently (50%) in specimens taken after gemcitabine-based (neo)adjuvant CTx (P=0.02). In multivariate analysis, ABCG2 expression was associated with an improved PFS (HR=0.64; 95%CI 0.43-0.94 (P=0.02)) of treatment with GemNab. Prior CTx, both in the (neo)adjuvant and palliative setting, was associated with shorter PFS of GemNab (P=0.03), and ABCG2 expression tended to correlate with improved PFS in these (P=0.07), but not in CTx-naïve patients (P=0.20). Similarly, a high ABCG2 expression was associated with improved overall survival (OS) only in patients with prior exposure to CTx (P=0.03). No associations of ABCG2 expression with CTx dosing or response rates were found.

**Conclusion:**

We found indications of upregulation of ABCG2 expression in tumors of patients previously exposed to gemcitabine, and ABCG2 expression correlated with efficacy of GemNab as assessed by PFS and OS in patients previously exposed to CTx, but not in those naïve to CTx. These findings diverge from the prevailing assumption that ABCG2 confers chemoresistance and suggest that in certain contexts, ABCG2 expression may reflect tumor adaptation or selection. Given the unexpected direction of this association, our findings should be interpreted as hypothesis-generating, and further studies are needed to elucidate underlying biological mechanisms and validate ABCG2 as a potential predictive biomarker in this setting.

## 1 Introduction

Pancreatic cancer (PC) is currently the sixth most common cause of cancer death (1), and is expected to become the second leading cause of cancer-related deaths in ten years (2, 3). The prognosis is grave as non-resectable disease is fatal, and surgery with curative intent can be offered to only 20-25% of patients (4).

Patients in good general condition with non-resectable disease are treated with chemotherapy (5). Compared to single-drug treatment with gemcitabine (Gem), the first drug approved for treatment of PC (6), combination-chemotherapy with folfirinox (5-flouroucil/leucovorin, irinotecan and oxaliplatin) or gemcitabine and *nab*-paclitaxel (GemNab) (7, 8) showed improved survival results. These regimens are now universally introduced as treatments of choice (5), GemNab being considered the most tolerable regime (9). Recently, the three-drug combination, Nalirifox (5-flouroucil/leucovorin, oxaliplatin and liposomal irinotecan) was shown to increase median overall survival by 2.9 months compared to GemNab, however, at a price of more toxicity (10, 11).

Drug resistance is a major limitation to the sustained effect of chemotherapy in PC (4, 12). Most tumors rapidly progress despite initial response, and many are clinically resistant from the start (4, 13). For example, in the pivotal MPACT study of GemNab, 20% of patients experienced progressive disease (PD) at the first evaluation and half of patients had progressed at 5.5 months (8). Therefore, several preclinical and clinical studies have investigated mechanisms of drug resistance relevant for the GemNab combination, but the mechanisms are still unclear (14).

Being a taxane, *nab*-paclitaxel (Nab) targets tubulin and stabilizes the microtubules causing cell cycle arrest and hence apoptosis (15, 16). The microtubule network consists of polymers of α- and β-tubulin and resistance may occur through aberrant expression of the isotype βIII-tubulin (17, 18). Other possible mechanisms of resistance include alterations in the tumor microenvironment and increased metabolism of the drug (4, 19, 20). Gem is a deoxycytidine nucleoside analogue and functions by inhibiting the DNA synthesis and by inhibiting progression of the cell cycle to G1/S-phase (21–23). Resistance to Gem may result from downregulation of nucleoside transporters, activation of cancer stem cells, epithelial-mesenchymal transition or inactivation of pathways for apoptosis (3, 15, 22).

For many chemotherapeutics, including Gem and taxanes, upregulation of drug efflux pumps may play an important role for treatment resistance (20, 21). By gaining energy from ATP hydrolysis, transmembrane efflux pumps including ATP-binding cassette (ABC) proteins can pump molecules against their gradient across plasma and intracellular membranes (24, 25). ABC-proteins are divided into seven subgroups (ABCA-ABCG) and are normally present in several kinds of tissues, such as the kidney, brain, pancreas and ovaries (24). The ABC proteins ABCB1 and ABCG2 have both been suggested as mediators of resistance to chemotherapy in PC (26) and in several other tumors (26–29). ABCB1 is known to efflux paclitaxel among other drugs but seems to be rarely expressed in PC (30, 31). ABCG2 (also called breast cancer resistance protein (BCRP)) regulates the uptake and removal of both internal and external substances, forms protective tissue barriers, and ensures the balance and stability of the body´s physiological systems (32). ABCG2 has been demonstrated to be significantly upregulated in PC tumors compared to non-malignant tissue (33). In PC cells *in vitro*, Gem causes upregulation of ACBG2 protein levels, increased levels of ABCG2 mRNA in PC cells are associated with acquired resistance to Gem, and Wnt5a-induced ABCG2 expression causes resistance to Gem (33–35). While neither Gem nor Nab are considered to be substrates (36), ABCG2 seems to be involved in the cellular response to oxidative stress, which is involved in initiation and progression of PC (24, 37), and may cause resistance to Gem by indirect mechanisms (38, 39). In addition, certain variant ABCG2 alleles are associated with increased risk of toxicity to taxanes (40). Clinical studies targeting ABC proteins are underway attempting to delay or prevent resistance to chemotherapy, including studies of drugs targeting ABCB1 and ABCG2 (29, 41).

The aim of this study was to determine the expression of ABCG2 and ABCB1 proteins in tumors from patients with PC, and to study the impact of these biomarkers on efficacy and outcome of treatment with GemNab.

## 2 Materials and methods

### 2.1 Patients

Through local registers, we identified 208 consecutive patients, more than 18 years of age, who initiated palliative treatment with GemNab for histologically confirmed, non-resectable PC from January 1^st^, 2011, to December 1^st^, 2019, at Aalborg University Hospital and Aarhus University Hospital, Denmark. Patients who had not received at least one CT scan after treatment initiation were excluded (N=40), to ensure that all patients were exposed to efficient doses of chemotherapy and could be evaluated for response. One patient was excluded due to missing clinical data. Finally, 28 cases were excluded due to insufficient archival tissue for immunohistochemistry (IHC). For the final study population of 140 patients, medical charts were reviewed for demographic, pathological and clinical data.

### 2.2 Treatment

GemNab was dosed and dose-adjusted according to manufactures prescription (42, 43). Actual dosing of Gem and Nab was registered in detail, and to assess the correlation between expression of ABC proteins in tumors and dosing of GemNab, delivered total dose (DTD), delivered dose intensity (DDI) and relative dose (RD) was calculated for each compound (41). Evaluation of treatment efficacy was performed every 8^th^ week by CT-scans supplemented by clinical and biochemical evaluation.

### 2.3 Pathological assessment

Tumor presence was confirmed in archived formalin-fixed, paraffin-embedded (FFPE) biopsy and surgical specimens from both primary tumors and metastases when available. Only tissue obtained prior to chemotherapy with GemNab was considered. New 5-µm sections were cut and deparaffinization was performed for 12 min. at 72°C, followed by heat-induced target retrieval at 97°C for 56 min. for ABCG2, and 44 min. for ABCB1. Both antibodies were visualized using Ventana Optiview DAB detection kit.

ABCG2 was detected by IHC using the Ventana Benchmark Ultra platform (Ventana Medical Systems, Tucson, AZ) Ventana IHC-DAB program. A rabbit monoclonal anti-ABCG2 antibody (Cat No. ab207732, RabMab EPR20080, AbCam) was diluted to 1:300 and incubated in 32 min. Normal human liver served as the positive control, and human placenta as the negative control. ABCB1 was identified using a rabbit monoclonal anti-P-Glycoprotein/ABCB1 antibody (Cat No. Ab170904; RabMab EPR10365-57, Abcam) applied at a 1:100 dilution and incubated in 40 min.

**Figure 1:**
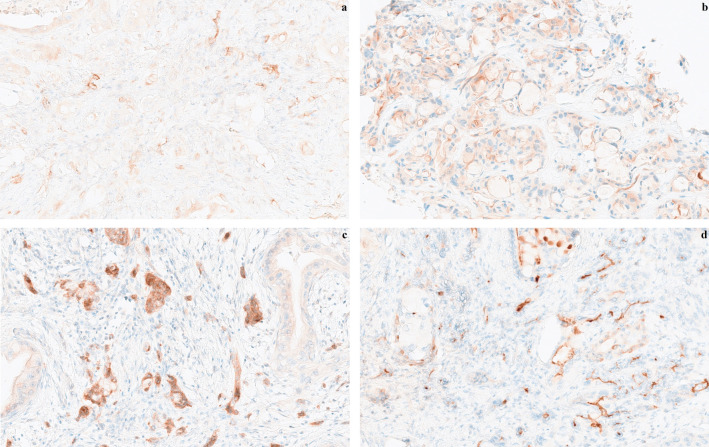
Representative histological sections of pancreatic cancers showing ABCG2 expression in tumors at 40x magnification, **(a)** score 1, **(b)** score 2, and **(c)** score 3, and **(d)** ABCB1 expression score 2 at 40x magnification. The scoring protocol for ABCG2 (ATP-binding cassette protein G2); was modified from Cederbye et al. (44): Score 0, basolateral membrane staining in less than 0-10% of tumor cells; score 1, weak basolateral membrane staining in at least 10% of tumor cells; score 2, weak to moderate basolateral membrane staining in at least 10% tumor cells; score 3, strong basolateral membrane staining in at least 10% of tumor cells. The scoring protocol for ABCB1 (ATP-binding casette protein B1) was based on H-score (45, 46).

ABCG2 and ABCB1 were independently evaluated by two pathologists, blinded to the treatment outcomes. Discrepancies were discussed to reach a consensus. In 123 patients one section was assessed, while two sections from different specimens were evaluated in 17 cases. For ABCG2, the scoring protocol was based on validated guidelines from Cederbye et al. (44). This approach specifically assesses membrane-associated staining, disregarding cytoplasmic staining unless accompanied by basolateral involvement. For ABCB1, no validated scoring system could be identified and the scoring system used was the semi-quantitative H-score (Histoscore) as previously described (45, 46). Representative sections with different scores for ABCG2 and the ABCB1 positive specimen are shown in **Figure 1**.

### 2.4 Endpoints

The primary endpoint was progression free survival (PFS) according to tumor tissue expression of ABC proteins. Secondary endpoints were overall survival (OS) and response to treatment. Response was assessed by review of medical charts by RECIST version 1.1 (47) in 119 (83%) patients with evaluable and measurable disease at baseline.

### 2.5 Ethics

The study was approved by the Regional Committee on Health Research Ethics in Northern Denmark (N-20200049) and by the Department of Research and Statistics in Northern Denmark (ID 2020-120). Permission was given to waiver patients’ consent for the study, however, permit for genotyping of patients was not provided.

### 2.6 Statistical analysis

All statistical analyses were performed using R version 4.4.2 (48). Index date was the start date of treatment with GemNab. Baseline characteristics of ABCG2 positive vs negative patients were compared using t-test or Fishers exact test for continuous and categorical variables, respectively. Kaplan-Meier curves were plotted for both PFS and OS and differences in survival were tested using a log-rank test. Multivariate survival analysis was performed using a Cox proportional hazed model adjusted for age, gender, performance status, clinical stage, prior palliative chemotherapy, and prior (neo)adjuvant chemotherapy. Cox regression was applied in both the entire cohort and in subgroups of, respectively, chemo-naïve, and previously treated patients. Survival analysis was done using the R package *survival* v3.7–0 and visualized using the package *survminer* v0.5.0 (49, 50).

## 3 Results

The study population consisted of a total of 140 patients, 77 included at Aalborg University Hospital and 63 at Aarhus University Hospital. The follow-up was almost complete, as all except three patients died. As shown in **Table 1**, column 1, the mean age was 65 years, and the majority were males. 83% had distant metastatic disease. A total of 81% were in ECOG PS 0-1, and 60% of patients received treatment with GemNab as 1^st.^ line of palliative chemotherapy. Forty patients had recurrent disease after pancreatic resection of which 35 (88% of those resected) had received neoadjuvant and/or adjuvant chemotherapy. A total of 46% of patients were naïve to any chemotherapy.

**Table 1:** Baseline characteristics of 140 patients with non-resectable pancreatic cancer treated with gemcitabine and *nab*-paclitaxel, distributed according to tumor expression of ABCG2.

Variable	All patients (n=140)	ABCG2 negative (n=94)	ABCG2 positive (n=46)	P-value*
Gender				
Female	52 (37%)	36 (38%)	16 (35%)	0.71
Male	88 (63%)	58 (62%)	30 (65%)	
Age (years)				
Mean (SD)	65 (9%)	65 (9%)	66 (9%)	0.41
ECOG PS				
0	22 (16%)	13 (14%)	9 (20%)	0.44
1	91 (65%)	61 (65%)	30 (65%)	
2	26 (19%)	20 (21%)	6 (13%)	
Missing	1 (1%)	0 (0%)	1 (2%)	
Tobacco				
Smoker	25 (18%)	18 (19%)	7 (%15)	0.28
Non-smoker	22 (16%)	13 (14%)	9 (20%)	
Former smoker	46 (33%)	35 (37%)	11 (24%)	
Unknown	47 (34%)	28 (30%)	19 (41%)	
BMI				
<18.5	9 (6%)	7 (7%)	2 (4%)	0.03
18.5-24.9	78 (56%)	57 (61%)	21 (46%)	
25-29.9	36 (26%)	24 (26%)	12 (26%)	
≥30	17 (12%)	6 (6%)	11 (24%)	
Clinical stage				
Metastatic	116 (83%)	75 (80%)	41 (89%)	0.23
Locally advanced	24 (17%)	19 (20%)	5 (11%)	
Primary tumor site				
Caput	66 (47%)	43 (46%)	23 (%50)	0.20
Cauda	25 (18%)	19 (20%)	6 (13%)	
Corpus	35 (25%)	25 (27%)	10 (22%)	
Papillar	9 (6%)	6 (6%)	3 (7%)	
Unknown	5 (4%)	1 (1%)	4 (9%)	
Serum Ca 19-9				
Non-expression	18 (13%)	14 (15%)	4 (9%)	0.44
Elevated (median 805 units/ml)	95 (68%)	63 (67%)	32 (70%)	
Unknown	27 (19%)	17 (18%)	10 (22%)	
Tumor type				
Adenocarcinoma	134 (96%)	91 (97%)	43 (94%)	0.42
Adenocarcinoma variants	7 (4%)	4 (4%)	3 (6%)	
Tissue origin				
Metastasis	49 (35%)	30 (32%)	19 (41%)	0.51
Primary tumor	75 (54%)	53 (56%)	22 (48%)	
Combined	15 (11%)	11 (12%)	4 (9%)	
Unknown	1 (1%)	0 (0%)	1 (2%)	
Specimen type				
Biopsy	104 (74%)	77 (82%)	27 (59%)	0.04
Resected specimen	30 (21%)	15 (16%)	15 (33%)	
Combined	3 (2%)	2 (2%)	1 (2%)	
Unknown	3 (2%)	0 (0%)	3 (7%)	
Prior (neo)adjuvant CTx				
Yes	35 (25%)	19 (20%)	16 (35%)	0.095
No	105 (75%)	75 (80%)	30 (65%)	
Prior (neo)adjuvant Gem-based CTx				
Yes	27 (19%)	13 (14%)	14 (30%)	0.02
No	113 (81%)	81 (86%)	32 (70%)	
Prior palliative CTx				
Any	56 (40%)	35 (37%)	21 (46%)	0.36
None	84 (60%)	59 (63%)	25 (54%)	
Any prior CTx				
Yes	75 (54%)	46 (49%)	29 (63%)	0.15
No (CTx naïve)	65 (46%)	48 (51%)	17 (37%)	

ABCG2, ATP-binding cassette protein G2; CA, cancer antigen; CTx, chemotherapy; ECOG PS, Eastern Cooperative Oncology Group performance status; BMI, body mass index; GemNab; gemcitabine and *nab*-paclitaxel; (neo)adjuvant, neoadjuvant and/or adjuvant; SD, standard deviation.

*Positive versus negative.

Only one patients’ tumor (0.7%) expressed ABCB1. This tumor also scored positive for ABCG2. Positive and negative controls had expression of ABCB1 and ABCG2 as expected.

Forty-six patients (33%) had tumor expression of ABCG2, of which 22 had a score of 1, 19 of 2 and five of 3. Positive and negative controls had expression of ABCG2 as expected. The heterogeneity in scores obtained in different biopsies from the same patient was considerable. In 17 patients with more than one biopsy assessed, the same score (negative versus positive) was given to both of two available sections in eight cases, while in nine cases one section was scored negative and one positive.

**Table 1**, column 2 and 3, shows the distribution of ABCG2 expression (negative versus positive) according to clinical and pathological factors. Increasing BMI was significantly associated with expression of ABCG2 (P=0.03), and patients that previously received (neo)adjuvant chemotherapy had a trend toward a higher frequency of ABCG2 expression (P=0.095). This association was statistically significant when only considering gemcitabine-based (neo)adjuvant regimens (P=0.02). When stratifying patients according to ABCG2 scores 0, 1 and 2–3 similar results were obtained (**Supplementary Table S1**). ABCG2 expression was significantly more frequent when assessed in resected tumor specimens (50%) compared to biopsies (26%) (P=0.04), while no difference was found comparing frequencies of ABCG2 expression in primary tumors (29% positive) or metastases (39% positive) (P=0.51).

In 122 patients evaluable for response assessment by RECIST criteria, the overall response rate (ORR) was 17%. All responses were partial. ABCG2 expression had no impact on ORR; 14 (17%) of 82 patients with no expression had a response while seven (18%) of 40 patients with ABCG2 expression responded (P=0.84).

**Figure 2:**
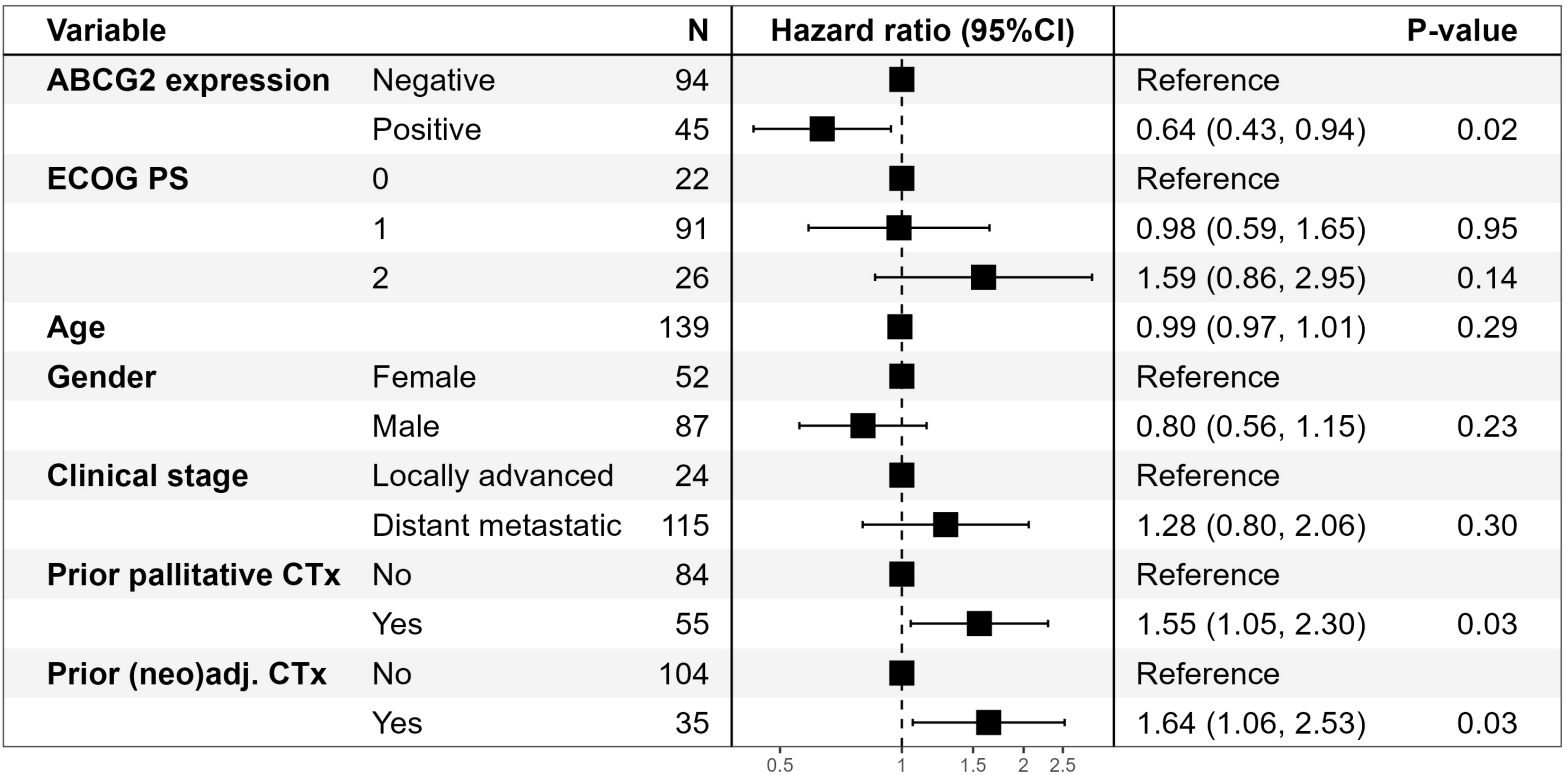
Forrest plot of results of multivariate analysis of PFS according to ABCG2 expression in 139* patients with non-resectable pancreatic cancer treated with gemcitabine and *nab*-paclitaxel. *One patient is excluded from the multivariate analysis due to missing performance status. ABCG2, ATP-binding cassette protein G2; CI, confidence interval; CTx, chemotherapy; ECOG PS, Eastern Cooperative Oncology Group performance status; (neo)adj., neoadjuvant and/or adjuvant.

Although there was a trend toward improved PFS outcome for patients with tumors expressing ABCG2, the primary endpoint was not reached in univariate analysis of the full cohort (P=0.08), and ABCG2 expression was not correlated with OS (P=0.93). At multivariate analysis, however, expression of ABCG2 was significantly associated with a longer PFS (HR=0.64; 95%CI 0.43-0.94 (P=0.02)), while prior palliative chemotherapy or prior (neo)adjuvant chemotherapy was associated with shorter PFS (P=0.03 in both cases). A Forrest plot of results is shown in **Figure 2**. In multivariate analysis according to OS, metastatic disease was the only factor approaching statistical significance (P=0.06) (**Supplementary Figure S2**). A supplementary multivariate analysis of PFS and OS using three strata for ABCG expression showed similar results (**Supplementary Figure S3**).

**Figure 3:**
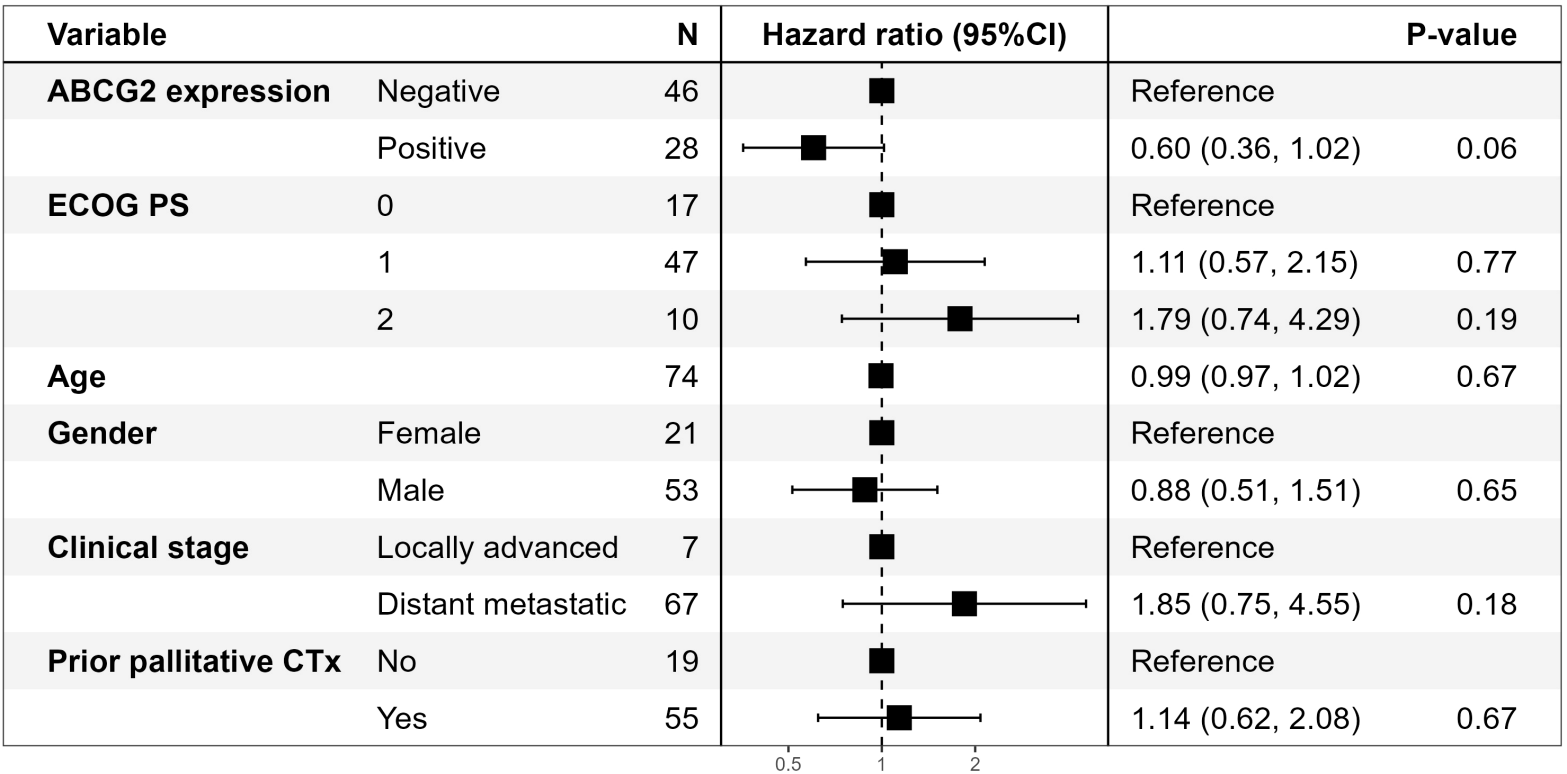
Forrest plot of results of multivariate analysis of PFS on treatment with gemcitabine and *nab*-paclitaxel according to ABCG2 expression in 74* patients with non-resectable pancreatic cancer previously treated with chemotherapy. *One patient is excluded from the multivariate analysis due to missing performance status.

As prior chemotherapy may impact ABCG2 expression in tissue, we made a stratified analysis of 65 patients that were naïve to chemotherapy and 75 patients that had previously been exposed to chemotherapy. ABCG2 expression tended to correlate with improved PFS of GemNab only in patients with prior exposure to chemotherapy (P=0.07), but not in chemo-naïve (P=0.20). In multivariate analysis of patients previously exposed to chemotherapy, the association of ABCG2 expression with longer PFS and OS of GemNab was nearly significant (P=0.06 in both cases). A Forrest plot of results according to PFS is shown in **Figure 3**. High ABCG2 expression (score 2-3) was significantly associated with longer OS (P=0.03) in patients previously exposed to chemotherapy (**Supplementary Figure S3**).

The association of tumor ABCG2 expression (negative versus positive) with delivered treatment is shown in **Table 2**. The median relative dose (RD) of Gem was 86% but only 73% for Nab. The delivered total dose (DTD) was higher for both drugs, and the RD of Nab slightly lower in ABCG2 positive patients, however, none of the differences were statistically significant (P≥0.18).

**Table 2:** Dose of chemotherapy according to ABCG2 expression in tumors of 140 patients with non-resectable pancreatic cancer treated with gemcitabine and *nab*-paclitaxel.

Dose variable	Value (Median, Range)	All patients (n=140)	ABCG2 expression		
			Negative (n=94)	Positive (n=50)	P-value
DTD of Gem (mg)	Median (range)	17,700 (3,600-71,000)	15,100 (3,600-71,000)	20,500 (3,600-53,000)	0.18
DDI^1^ of Gem (mg/day)	Median (range)	154 (18.3-571)	155 (18.3-571)	153 (76.3-280)	0.32
RD of Gem (%)^2^	Median (range)	85.5 (21.6-106)	85.5 (21.6-106)	85.4 (42-101)	0.57
DTD of Nab (mg)	Median (range)	1,470 (187-7,360)	1,370 (187-7,360)	1,840 (384-6,120)	0.65
DDI^1^ of Nab (mg/day)	Median (range)	16.6 (1.1-71.4)	16.7 (1.5-71.4)	16.6 (1.1-27.7)	0.20
RD of Nab (%)^3^	Median (range)	72.8 (4.3-106)	73.3 (6.9-106)	70.4 (4.3-100)	0.39

1) DDI = DTD/days on treatment.

2) RD of Gem = DTD/(BSA x 1,000 mg x number of treatments) x 100%.

3) RD of Nab = DTD/(BSA x 125 mg x number of treatments) x 100%.

ABCG2, ATP-binding cassette protein G2; BSA, estimated body surface area (in m^2^); DDI, delivered dose intensity; DTD, delivered total dose; Gem, gemcitabine; Nab, *nab*-paclitaxel; RD, relative dose; BSA, body surface area.

## 4 Discussion

In this retrospective cohort study of patients with non-resectable PC, we examined whether tumor-tissue expression of drug efflux pumps ABCB1 and ABCG2 was associated with outcome of chemotherapy with gemcitabine and *nab*-paclitaxel. PFS was the primary endpoint. In our cohort, ABCB1 was expressed in only one (0.7%) of 140 patients. The frequency of expression of ABCB1 protein or mRNA in PC has been reported very differently in the literature, ranging from just a few percent to 72.8%. The variation may be caused by differences in methodology, tumor heterogeneity, observer variability in assessment, and patient-related factors such as patients’ ethnicity or prior treatment with chemotherapy (30, 51). In contrast, ABCG2 was expressed in 33% and, although only nearly significant in univariate analysis of the full cohort, ABCG2 expression was of significant, independent predictive value according to PFS of GemNab in multivariate analysis. The predictive value, which also included an association with improved OS for patients with high expression levels of ABCG2 was, however, confined to patients who had been exposed to chemotherapy prior to treatment with GemNab.

Most often, ABCG2 expression in cancers has been associated with poor prognosis, although results are inconsistent (52, 53), and in some an inverse association was found (38, 54). In 60 patients with intrahepatic cholangiocarcinoma a longer survival time was observed in patients with ABCG2 expression in moderately to poorly differentiated tumors, but not in well differentiated (54), and in an analysis within the Cancer Genome Atlas (TCGA) program, high expression levels of ABCG2 were associated with improved prognosis in adrenocortical carcinoma, glioblastoma and renal clear cell carcinoma (38). In PC, only a few prior studies have examined the expression of ABCG2 and its impact on prognosis. In a study by Lee et al., ABCG2 expression was found in 73% of 67 samples analyzed and high expression levels were associated with short time to progression and poor overall survival (55). Yuan et al. examined ABCG2 expression in 106 chemo-naïve patients with PC, and in 103 specimens of peritumoral tissue, benign pancreatic lesions, or normal pancreatic tissue (56). ABCG2 was more frequently expressed (58%) in cancer tissue compared to other lesions, and its expression correlated with low differentiation, metastatic disease, and poor prognosis.

In contrast to the above, we found a lower fraction of tumors that expressed ABCG2, and expression did not correlate with patients’ survival in the full cohort, Methodological differences among studies associated with, *e.g.*, antibody specificity, retrieval and staining may apply (57). Evaluation may be subject to inter-observer variation, although we used a validated protocol, and two observers determined the final score by mutual agreement. Diagnostic biopsies are small and targeted to non-necrotic areas and may be more peripheral. The tumor may be better oxygenated and may show less stromal context. Larger specimens may capture more of the tumor microenvironment, which is known to regulate transporter expression (15). We did not evaluate the stromal component and we did not assess the distribution of the expression in the specimens. The small biopsies may miss this, which could explain the lower positivity and unexpected association with outcome. Moreover, inter-lesional heterogeneity in scores was observed that may influence decision-making for possible treatment.

The finding that the fraction of ABCG2-positive was higher in resected specimens than in biopsies but similar in primary tumors and metastases, suggests that size of tumor area assessed may impact results. Representativeness of sections may be important as demonstrated, *e.g.*, when assessing HER2 protein expression in gastric cancers (58). The ethnicity of patients could also influence results as our study included a population of Caucasians, whereas most prior studies were done in Asian populations. Ethnic genetic variations in ABC transporters, for instance ABCG2 421C>A polymorphism have been demonstrated with variant alleles up to 34% in Chinese populations compared to 11-12% in North American and European Caucasians (59, 60). Such genetic differences may influence transporter activity and chemotherapy disposition (60) (61). Interestingly, certain alleles have been associated with obesity (62), possibly explaining the significant association of ABCG2 expression with BMI found in the present study.

The association of ABCG2 expression in tumors with efficacy of chemotherapy has been demonstrated *in vitro* but is poorly investigated *in vivo* (32, 63). In three studies of advanced non-small-cell lung cancer patients treated with platinum-based chemotherapy, expression of ABCG2 was not associated with ORR or PFS in two (64, 65), while in one study, ABCG2 expression was associated with short PFS and a numerically lower ORR (66). In PC, a prior study showed an association between high ABCG2 expression and early recurrence of patients treated with adjuvant gemcitabine-based chemotherapy but response to chemotherapy could not be assessed (55). A study published only in abstract form using RNA profiling of circulating tumor and invasive cells in 33 patients suggested that high ABCG2 gene expression was associated with shorter PFS on treatment with GemNab (67). In the current study, we found no indications of upregulated ABCG2 being associated with *de novo* chemo-resistance, response rate or poor outcome of GemNab. On the contrary, ABCG2 expression in samples from patients that had received chemotherapy in a (neo)adjuvant or first-line palliative setting, but prior to treatment with GemNab, was associated with an improved efficacy of GemNab as assessed by PFS. From this we hypothesize that tumors, where ABCG2 is upregulated during prior chemotherapy, are more sensitive to GemNab. Although the interpretation of this unexpected result is speculative and confirmatory studies are needed, ABCG2 expression could therefore be a biomarker for personalized selection of patients to later-line treatment with GemNab. This association may also have implications for clinical trials investigating ABCG2-inhibitors together with chemotherapy (41).

The selection of patients with best chance of response to GemNab is particularly important in those pretreated with chemotherapy as this population may benefit less. Although efficacy of GemNab in patients previously treated with (neo)adjuvant chemotherapy is poorly investigated and patients included in the pivotal randomized trial of GemNab were exclusively chemo-naïve (8), shorter PFS of GemNab in the 2^nd^ line, palliative setting has been shown in several reports (68, 69). In the current study, ABCG2 expression was not predictive of PFS of GemNab in chemo-naïve patients and therefore other mechanisms of early resistance as recently reviewed by Espona-Fiedler et al. (70), must be in play.

ABCG2 expression was more frequent in patients previously exposed to Gem in the curative setting in accordance with preclinical studies showing that Gem can induce upregulation of ABCG2 in PC cells (39). Finally, we found no significant association between ABCG2 expression and chemotherapy dosing, indicating that tolerability of both Gem and Nab was not associated with ABCG2 expression. Further evaluation of these aspects, including assessment of whether a potential predictive value is associated with Gem, Nab or both drugs, will be investigated in an ongoing randomized study (71).

Main limitations of this study are its retrospective design and lack of sufficient tissue in 17% of patients. Although all patients received GemNab in a palliative setting, the cohort was heterogeneous with respect to prior surgical and oncological treatment. This, however, allowed us to analyze the impact of prior chemotherapy on results. We were unable to assess effects of treatment on biomarker expression at the individual patient level as only 17 patients had paired biopsies taken before and after treatment. Further, we had no information on allele frequencies that may explain different outcomes in different populations. ABCG2 expression has been shown to correlate with epithelial-mesenchymal transition markers, which are known to interact with drug efflux pathways (23), and further studies are warranted to elucidate this aspect. We chose to exclude individuals not reaching their first evaluation scan (19%) to ensure that patients were exposed to reasonable doses of chemotherapy and could be evaluated for response. Although this introduces immortal time bias and bias toward more responsive and fitter patients being selected, our primary focus was to assess associations of biomarkers with respect to efficacy of chemotherapy, not the prognostic value. At the treating departments, folfirinox was the preferred 1^st^ line palliative treatment option for patients in good general condition (72) and, hence, the population was selected toward poor prognostic factors and comorbidities. Finally, methodological issues related to ABCG2 scoring as outlined above are poorly investigated and should be addressed, if ABCG2 expression is to be used as a clinical biomarker. Digital image analysis and quantitative IHC approaches are emerging and could be helpful in quantifying ABCG2 expression.

In conclusion, ABCG2 was expressed in approximately one third of tumors from patients treated with GemNab. Among patients previously exposed to chemotherapy, ABCG2 expression showed a potential association with improved efficacy of GemNab. While these results are intriguing, they contrast with prior reports linking ABCG2 with drug resistance, and the biological rationale for this inverse association remains unclear. Thus, these findings should be considered hypothesis-generating, and further studies are warranted to explore possible mechanisms—such as adaptive stress responses, clonal selection, or microenvironmental factors—that may contribute to this observation.

## Data Availability

The raw data supporting the conclusions of this article will be made available by the authors, without undue reservation.
