# Supplementary material for: ABCG2 protein expression in tumors of patients with non-resectable pancreatic cancer treated with gemcitabine and nab-paclitaxel
Source: Front Oncol. 2025 Jun 6;15:1558184. doi: 10.3389/fonc.2025.1558184 (PMC12178868; doi:10.3389/fonc.2025.1558184)
Supplement: Supplementary file 1 [file DataSheet1.docx]

**Supplementary tables and figures (ABC)**

**Supplementary table 1S. Baseline characteristics of 140 patients with non-resectable pancreatic cancer treated with gemcitabine and *nab*-paclitaxel, distributed according to ABCG2 scores.**

|  | **ABCG2 expression** | | | **P-value** |
| --- | --- | --- | --- | --- |
|  | **Score 0 (n=94)** | **Score 1**  **(n=22)** | **Score 2-3(n=24)** |  |
| **Gender** |  |  |  |  |
| Female | 36 (38%) | 7 (32%) | 9 (37%) | 0.48 |
| Male | 58 (62%) | 15 (68%) | 15 (63%) |  |
| **Age** (years) |  |  |  |  |
| Mean (SD) | 65 (9) | 67 (7) | 65 (10) | 0.84 |
| Median (Min, Max) | 66 (39, 80) | 68 (51, 80) | 68 (42, 80) |  |
| **ECOG PS*** |  |  |  |  |
| 0 | 13 (14%) | 2 (9%) | 7 (29%) | 0.14 |
| 1 | 61 (65%) | 14 (64%) | 16 (67%) |  |
| 2+ | 20 (21%) | 5 (23%) | 1 (4%) |  |
| Missing | 0 (0%) | 1 (4%) | 0 (0%) |  |
| **Tobacco** |  |  |  |  |
| Smoker | 18 (19%) | 3 (14%) | 4 (17%) | 0.39 |
| Non-smoker | 13 (14%) | 6 (27%) | 3 (13%) |  |
| Former smoker | 35 (37%) | 6 (27%) | 5 (21%) |  |
| Unknown | 28 (0%) | 7 32%) | 12 (50%) |  |
| **BMI** |  |  |  |  |
| <18.5 | 7 (7%) | 1 (5%) | 1 (4%) | 0.72 |
| 18.5-24.9 | 57 (61%) | 8 (36%) | 4 (54%) |  |
| 25-29.9 | 24 (26%) | 7 (32%) | 5 (21%) |  |
| ≥30 | 6 (6%) | 6 (27%) | 5 (21%) |  |
| **Clinical stage** |  |  |  |  |
| Metastatic | 75 (80%) | 18 (82%) | 23 (96%) | 0.18 |
| Locally advanced | 19 (20%) | 4 (18%) | 1 (84%) |  |
| **Primary tumor site** |  |  |  |  |
| Caput | 43 (46%) | 11 (50%) | 12 (50%) | 0.31 |
| Cauda | 19 (20%) | 2 (9%) | 4 (17%) |  |
| Corpus | 25 (27%) | 6 (27%) | 4 (17%) |  |
| Papillar | 6 (6%) | 2 (9%) | 1 (4%) |  |
| Unknown | 1 (1%) | 1 (5%) | 3 (12%) |  |
| **Serum Ca 19-9** |  |  |  |  |
| Non-expression | 14 (15%) | 2 (9%) | 2 (8%) | 0.79 |
| Elevated (median 805 units/ml) | 63 (67%) | 15 (68%) | 17 (71%) |  |
| Unknown | 17 (18%) | 5 (23%) | 5 (21%) |  |
| **Tumor type** |  |  |  |  |
| Adenocarcinoma | 91 (97%) | 20 (91%) | 23 (96%) | 0.21 |
| Adenocarcinoma variants | 3 (3%) | 2 (9%) | 1 (4%) |  |
| **Tissue origin** |  |  |  |  |
| Metastasis | 30 (32%) | 9 (41%) | 10 (42%) | 0.85 |
| Primary tumor | 53 (56%) | 10 (46%) | 12 (50%) |  |
| Both | 11 (12%) | 2 (9%) | 2 (8%) |  |
| Unknown | 0 (0%) | 1 (4%) | 0 (0%) |  |
| **Specimen type** |  |  |  |  |
| Biopsy | 77 (82%) | 14 (64%) | 13 (54%) | 0.050 |
| Resected specimen | 15 16%) | 5 (23%) | 10 (42%) |  |
| Both | 2 (2%) | 0 (0%) | 1 (4%) |  |
| Unknown | 0 (0%) | 3 (14%) | 0 (0%) |  |
| **Prior (neo)adjuvant CTx** |  |  |  |  |
| Yes | 19 (20%) | 5 (23%) | 11 (46%) | 0.04 |
| No | 75 (80%) | 17 (77%) | 13 (54%) |  |
| **Prior (neo)adjuvant Gem** |  |  |  |  |
| Yes | 13 (14%) | 3 (14%) | 11 (46%) | 0.003 |
| No | 81 (86%) | 19 (86%) | 13 (54%) |  |
| **Prior palliative CTx** |  |  |  |  |
| None | 35 (37%) | 7 (32%) | 14 (58%) | 0.13 |
| Folfirinox | 59 (63%) | 15 (68%) | 10 (42%) |  |
| **Any prior CTx** |  |  |  |  |
| Yes | 46 (49%) | 12 (54%) | 17 (71%) | 0.16 |
| No (CTx naive) | 48 (51%) | 10 (46%) | 7 (29%) |  |

CTx, chemotherapy; ECOG PS: Eastern Cooperative Oncology Group performance status, BMI: Body Mass Index, GemNab; Gemcitabine and *nab*-paclitaxel.

**Supplementary figure 1S. Forrest plot illustrating results of multivariate analysis according to OS in 139* patients with non-resectable pancreatic cancer treated with gemcitabine and *nab*-paclitaxel.**

**
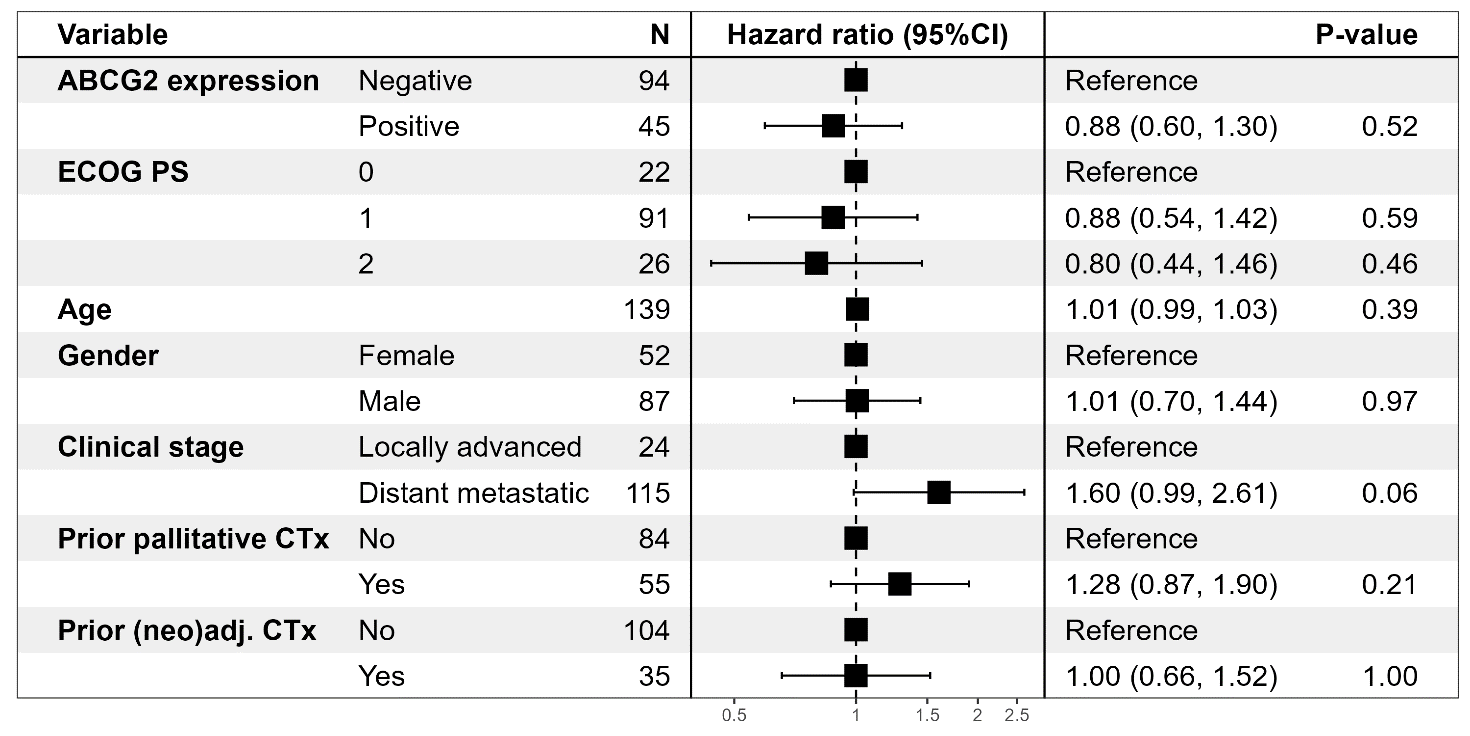
**

*****One patient is excluded from the multivariate analysis due to missing value in PS *****One patient is excluded from the multivariate analysis due to missing performance status.

**Supplementary figure 2S. Forrest plots of results of multivariate analysis according to, a) PFS, and b) OS in 139* patients with non-resectable pancreatic cancer treated with gemcitabine and *nab*-paclitaxel, distributed according to ABCG2 score 0, 1 and 2**

**a)**

**
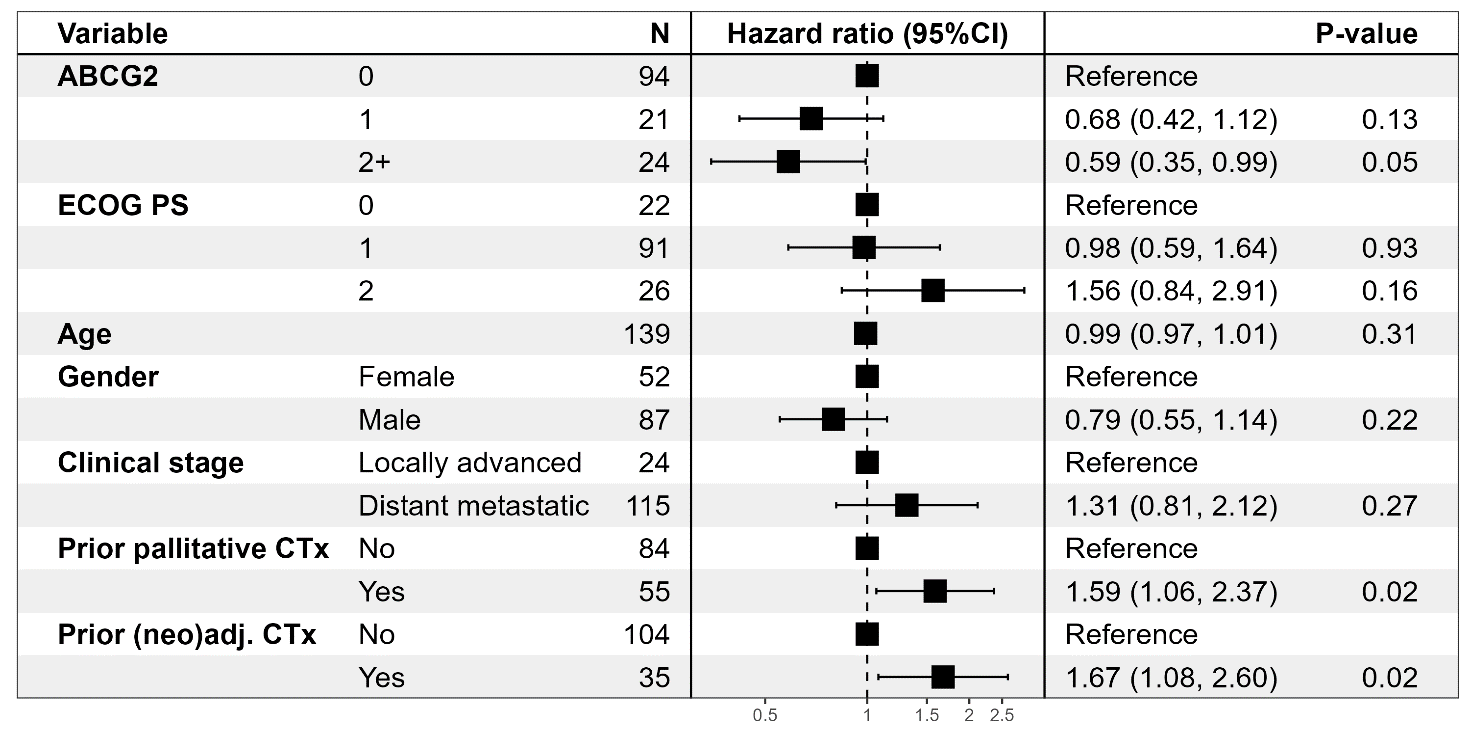
**

**b)**

**
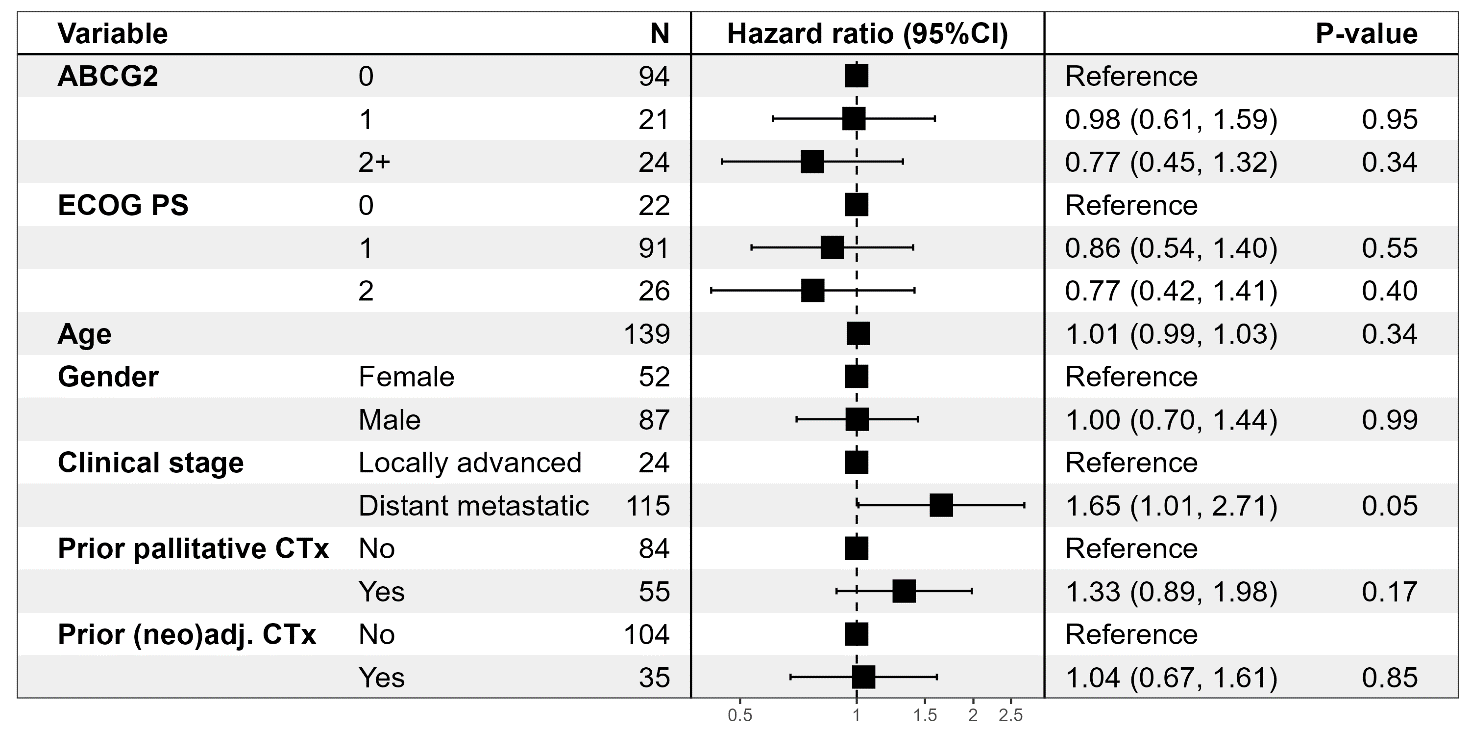
**

*****One patient is excluded from the multivariate analysis due to missing performance status.

**Supplementary figure 3S. Forrest plots of results of multivariate analysis according to, a) PFS, and b) OS in 74* patients with non-resectable pancreatic cancer treated with gemcitabine and *nab*-paclitaxel, who previously were treated with chemotherapy, distributed according to ABCG2 score 0, 1 and 2-3.**

**a)**

**
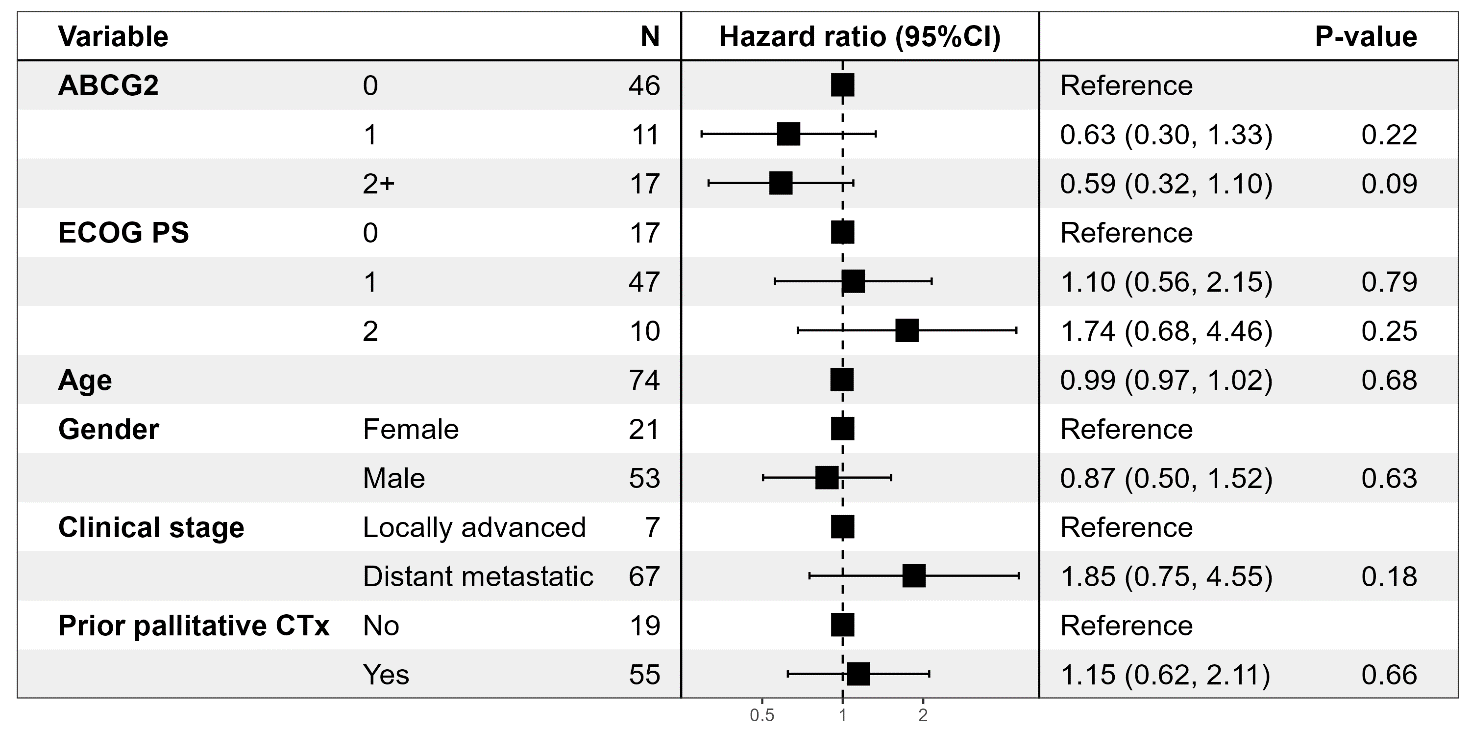
**

**b)**

**
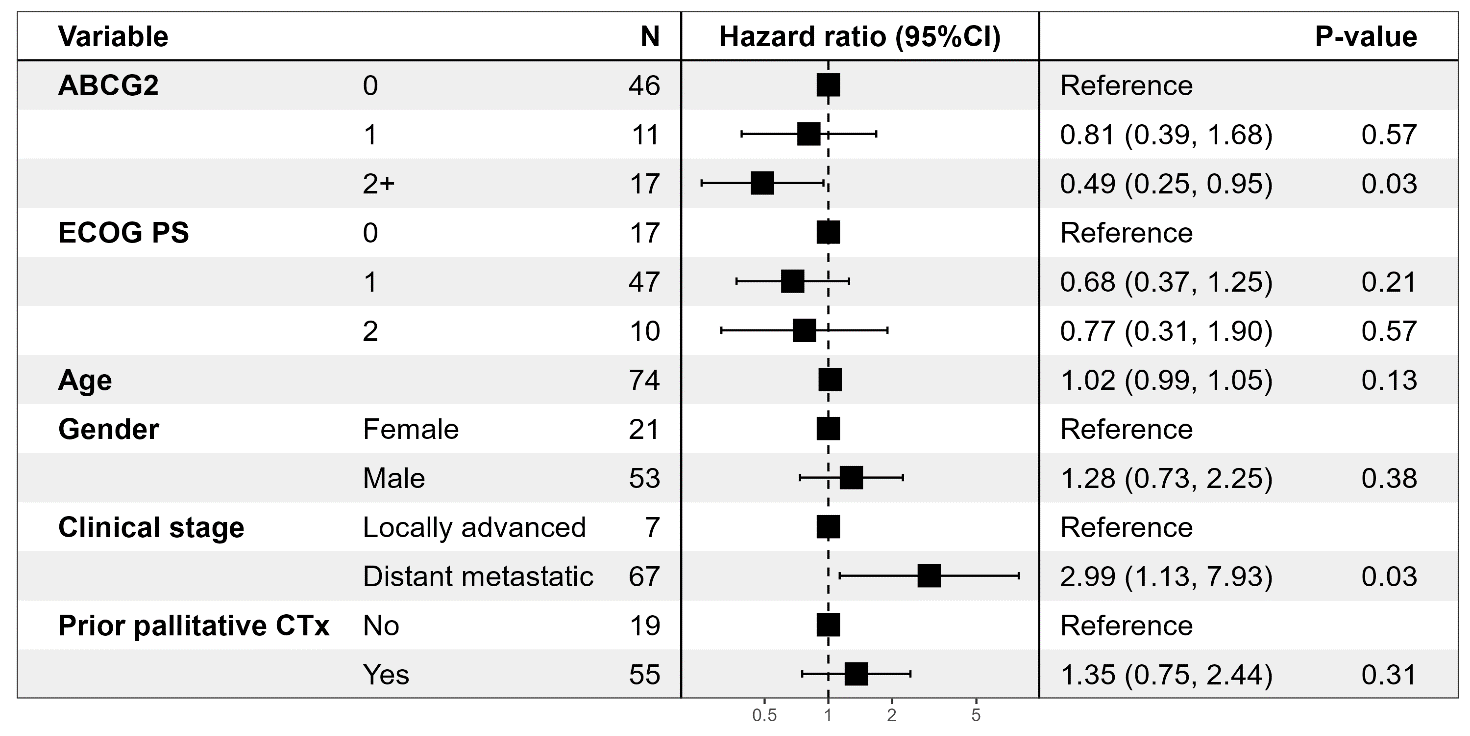
**

*****One patient is excluded from the multivariate analysis due to missing performance status.
